# Variability of HCC Tumor Diameter and Density Measurements on Dynamic Contrast-Enhanced Computed Tomography

**DOI:** 10.3390/tomography11030036

**Published:** 2025-03-19

**Authors:** Siddharth Guha, Abdalla Ibrahim, Pengfei Geng, Qian Wu, Yen Chou, Oguz Akin, Lawrence H. Schwartz, Chuan-Miao Xie, Binsheng Zhao

**Affiliations:** 1 Department of Radiology, Columbia University Irving Medical Center, New York, NY 10032, USA; guhas2@nychhc.org (S.G.); yc4065@cumc.columbia.edu (Y.C.); 2 Department of Radiology, Memorial Sloan Kettering Cancer Center, New York, NY 10065, USA; ibrahia1@mskcc.org (A.I.); gengp@mskcc.org (P.G.); wuq1@mskcc.org (Q.W.); akino@mskcc.org (O.A.); schwartzl@mskcc.org (L.H.S.); 3 Sun Yat-sen University Cancer Center, Guangzhou 510060, China; xiechm@sysucc.org.cn

**Keywords:** contrast-enhanced CT, hepatocellular carcinoma, lesion density

## Abstract

Purpose: In cancers imaged using contrast-enhanced protocols, such as hepatocellular carcinoma (HCC), formal guidelines rely on measurements of lesion size (in mm) and radiographic density (in Hounsfield units [HU]) to evaluate response to treatment. However, the variability of these measurements across different contrast enhancement phases remains poorly understood. This limits the ability of clinicians to discern whether measurement changes are accurate. Methods: In this study, we investigated the variability of maximal lesion diameter and mean lesion density of HCC lesions on CT scans across four different contrast enhancement phases: non-contrast-enhanced phase (NCE), early arterial phase (E-AP), late arterial phase (L-AP), and portal venous phase (PVP). HCC lesions were independently segmented by two expert radiologists. For each pair of a lesion’s scan timepoints, one was selected randomly as the baseline measurement and the other as the repeat measurement. Both absolute and relative differences in measurements were calculated, as were the coefficients of variance (CVs). Analysis was further stratified by both contrast enhancement phase and lesion diameter. Results: Lesion diameter was found to have a CV of 5.11% (95% CI: 4.20–6.01%). About a fifth of the measurement’s relative changes were greater than 10%. Although there was no significant difference in diameter measurements across different phases, there was a significant negative correlation (R = −0.303, *p*-value = 0.030) between lesion diameter and percent difference in diameter measurement. Lesion density measurements varied significantly across all phases, with the greatest relative difference of 47% in the late arterial phase and a CV of 22.84% (21.48–24.20%). The overall CV for lesion density measurements was 26.19% (24.66–27.72%). Conclusions: Changes in tumor diameter measurements within 10% may simply be due to variability, and lesion density is highly sensitive to contrast timing. This highlights the importance of paying attention to these two variables when evaluating tumor response in both clinical trials and practice.

## 1. Introduction

Computed tomography (CT) scans play a crucial role in both oncologic clinical practice and research, guiding clinicians in assessing cancer response to therapy and providing insight into tumor growth over time [1]. As software for viewing and segmenting tumors continues to advance, radiologists can now obtain increasingly accurate quantitative assessments of tumor features, such as diameter, area, volume, and density. These metrics are frequently employed in the evaluation of novel therapies in clinical trials. For example, the Response Evaluation Criteria In Solid Tumors (RECIST version 1.1) define partial response and progressive disease as a 30% or more decrease and a 20% or more increase in the sum of maximal in-plane diameters of target lesions, respectively, with stable disease referring to any change within this range [2]. This guideline has been well established as the gold standard in tumor response assessment and is even recommended by the Food and Drug Administration (FDA) for use as a clinical trial endpoint for the development of cancer drugs and biologics [3].

However, a major challenge in the use of these criteria is that tumor measurements are treated as categorical rather than continuous variables. Because Phase II clinical trials of novel therapies increasingly report continuous endpoints of tumor size change [4,5], it becomes difficult to fully assess the efficacy of these treatments using RECIST. Consider a theoretical trial in which treatment results in statistically significant tumor shrinkage of 25% in nearly all patients. According to RECIST, this would be classified as “stable disease” even though the therapy had a measurable impact on the tumor. Furthermore, many targeted molecular therapies and immunotherapies have been shown to exhibit strong therapeutic responses without substantial tumor shrinkage [6,7], or they may show pseudo-progression [8]. This underscores the need for a more nuanced interpretation of imaging measurements, particularly in understanding what changes might be clinically meaningful versus inherent measurement variability. Previous studies have shown that diameter measurements of lung cancer lesions can vary by as much as 10% for a single reader [9,10,11].

One potential source of measurement variability comes from the different levels of contrast enhancement of images, especially abdominal imaging, taken using a contrast-enhanced computed tomography (CECT) protocol. This type of imaging protocol is widely used in the diagnosis and assessment of treatment response of hepatocellular carcinoma (HCC), a primary liver tumor whose treatment response assessment can be inconsistent with traditional RECIST [7]. To address these inconsistencies, modified RECIST and modified Choi criteria incorporate tumor density [12]. Modified RECIST specifies that the change in diameter qualifying for disease progression or partial response must occur in a lesion that is “enhancing” in the arterial phase [13]. The modified Choi criteria go even further, stating that only a reduction in density of more than 15% and a reduction in diameter of more than 10% together qualify as a partial response to therapy, and any progressive disease must not meet the partial response criteria based on tumor density [14]. A decrease in lesion density alone has also been shown to be correlated with overall survival in HCC patients [15]. Although these criteria usually specify a contrast enhancement phase from which measurements are to be taken, the variability of measurements within each phase and between phases has yet to be thoroughly studied.

Moreover, the clinical implications of these measurement criteria are profound, as they directly influence patient outcomes and treatment strategies. For instance, if measurements are not reproducible or if variability is overlooked, a patient who could benefit from continuing therapy might be prematurely switched to a different treatment regimen. Conversely, misclassifying stable disease could lead to missed opportunities for more aggressive or alternative therapies. Such decisions underscore the importance of clear, consistent, and precise imaging-based assessment methods in guiding oncologic management.

Because each series of scans within a dynamic CECT protocol effectively functions as a repeat CT image for both lesion diameter and lesion density, in this study, we aimed to quantify the magnitude of variability of tumor size and density measurements within and across clinically defined contrast enhancement phases. Given that tumor density measurements for metastatic colorectal cancer can vary by as much as 15% within the portal venous phase [16], we sought to characterize the variability of lesion size and density both within and among the four different imaging phases. A more detailed understanding of this variability will not only aid radiologists in creating more reproducible measurements of tumor diameter and density for response assessment purposes but also help clinicians more effectively scrutinize the results of novel treatments in clinical trials.

## 2. Methods

### 2.1. Study Dataset

De-identified dynamic CECT scans from 68 patients who underwent liver lesion evaluation at a single medical center were retrospectively obtained under institutional review board approval. To be eligible, patients had to have (i) pathologically confirmed HCC and (ii) imaging data free of artifacts. Based on these criteria, 51 patients were included in the final analysis (Table 1). All scans were acquired before the initiation of treatment (Table 2).

This investigation was conducted in accordance with the Declaration of Helsinki and was approved by the Institutional Review Board of Sun Yat-sen University Cancer Center (SYSUCC) (protocol code 510060, approved 9 November 2022). The Institutional Review Board of SYSUCC waived the requirement for informed consent. Data were accessed for research purposes on 11 January 2023.

### 2.2. Reference Standard for Contrast Enhancement Phases

The reference standard used to stratify each patient’s scans consisted of labels of one of four contrast enhancement phases defined for dynamic CT-scan images: (I) non-contrast-enhanced phase (NCE), (II) early arterial phase (E-AP), (III) late arterial phase (L-AP), and (IV) portal venous phase (PVP). Labels for the reference study were assigned by radiologists based on the LI-RADS Version 2018 criteria for defining dynamic CT phases [17] and other commonly used clinical criteria [18,19,20]. To assign a label to each scan, two radiologists (P.G. and Q.W.) with four and five years of experience in abdominal imaging each independently assigned labels to each of the scans. Disagreements on phase labels were reviewed and discussed with a third radiologist (Y.C.) with six years of experience, and a consensus was reached on the label. The total number of scans assigned to each label was as follows: NCE—236, E-AP—127, L-AP—818, PVP—874.

### 2.3. Lesion Segmentation and Measurements

**Figure 1:**
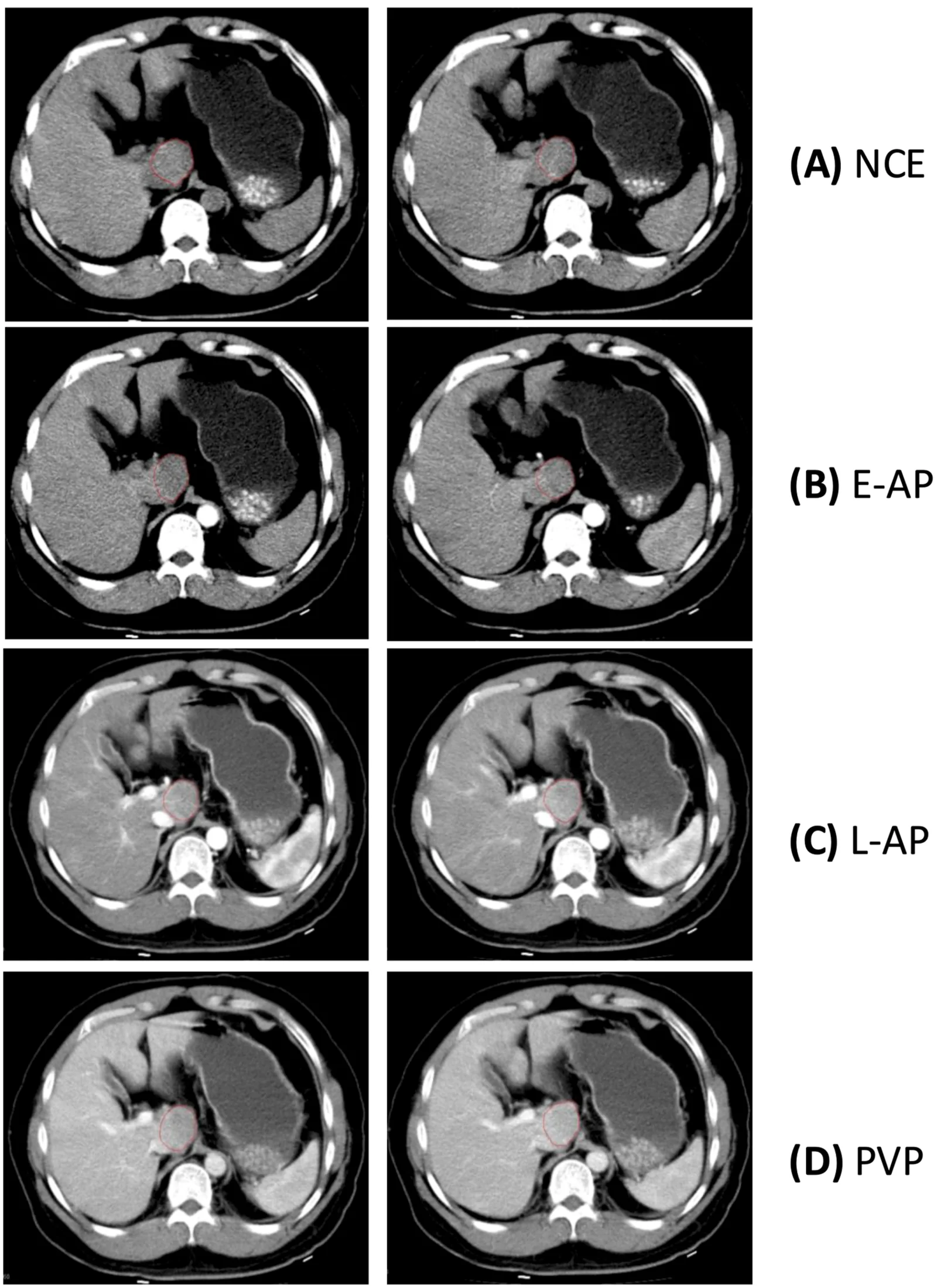
A lesion’s segmentations (circled red area) in the different phases.

The two radiologists, henceforth labeled radiologists 1 and 2, independently segmented the lesions for each patient by first drawing the lesion contour on the scan within the image series on which they best visualized the lesion for each patient. These segmentations were then copy–pasted across all the other scan timepoints for that patient and then edited to account for any variation due to respiration or patient movement (Figure 1). Lesion maximal in-plane diameter (measured in mm) and mean density (measured in Hounsfield units (HU)) were then extracted from each segmentation. The measurements between the two radiologists were averaged for both diameter and density when assessing the variability using pairwise comparisons. For descriptive statistics such as mean and standard deviation (SD), both radiologists’ measurements were pooled into one set.

### 2.4. Biostatistics

All analyses were conducted in Python (version 3.8) using the NumPy (2.2.0), Pandas (2.0.3), SciPy (1.11.1), Matplotlib (3.7.2), and Seaborn (0.12.2) packages. For each lesion, the baseline measurement was defined as the measurement from the initial segmentation sequence. Subsequent lesion measurements were compared to this baseline using the following formula for percent difference:

$$\mathrm{Percent}\;\mathrm{Difference}=\frac{\mathrm{Baseline}\;\mathrm{Value}-\mathrm{Measurement}}{\mathrm{Baseline}\;\mathrm{Value}}\times 100$$

Absolute differences were also computed as the simple difference between the baseline and each measurement.

Lesion measurements were averaged and aggregated on a per-lesion basis across radiologists. Specifically, for each segmented lesion, the coefficient of variance (CV), mean, and SD of the measurements were computed using a custom function. In addition, for each lesion, the maximum percent difference from baseline was determined, and “waterfall” plots were generated to visualize the distribution of maximum changes across lesions.

Lesion sizes were further stratified into size ranges (1–2 cm, 2–3 cm, 3–5 cm, and 5–7 cm), and the mean lesion size, CV, and corresponding SD were computed for each lesion per group. Pearson’s correlation coefficients were used to evaluate the relationship between lesion size and the percent difference in measurements.

**Figure 2:**
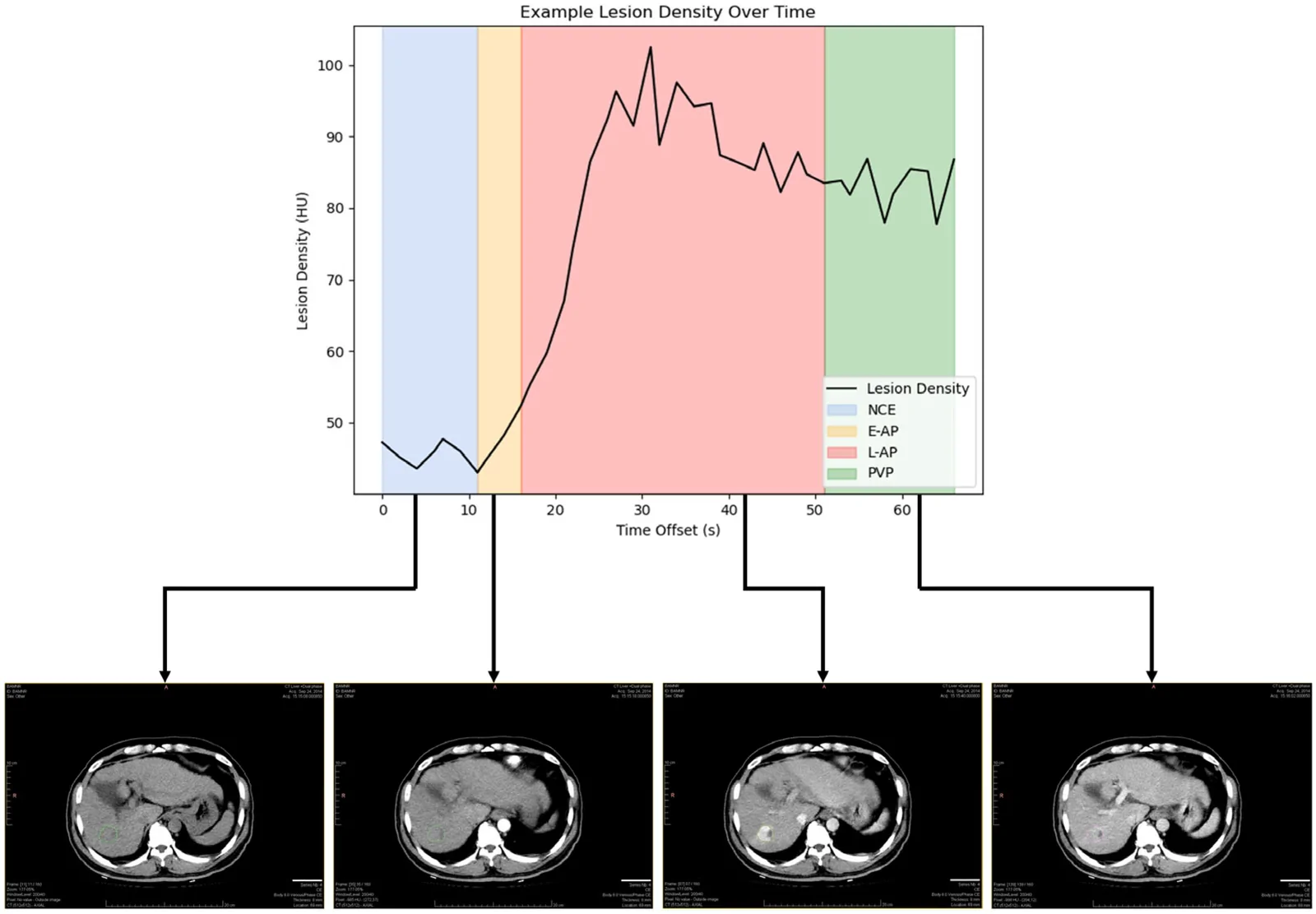
Density of HCC across contrast enhancement phases.

Given that lesion density can vary significantly across a dynamic CECT series (see Figure 2), the mean and SD of lesion density were calculated for each patient and compared across phases. Overall lesion density as well as phase-specific densities (NCE, E-AP, L-AP, and PVP) were computed and visualized using histograms and boxplots. In the phase-specific analysis, the mean density for each lesion was computed.

A two-tailed *p*-value of <0.05 was considered statistically significant. All descriptive statistics (means, standard deviations, and 95% confidence intervals) are reported separately for each radiologist.

## 3. Results

### 3.1. Variability of Diameter Measurement by Phase

**Figure 3:**
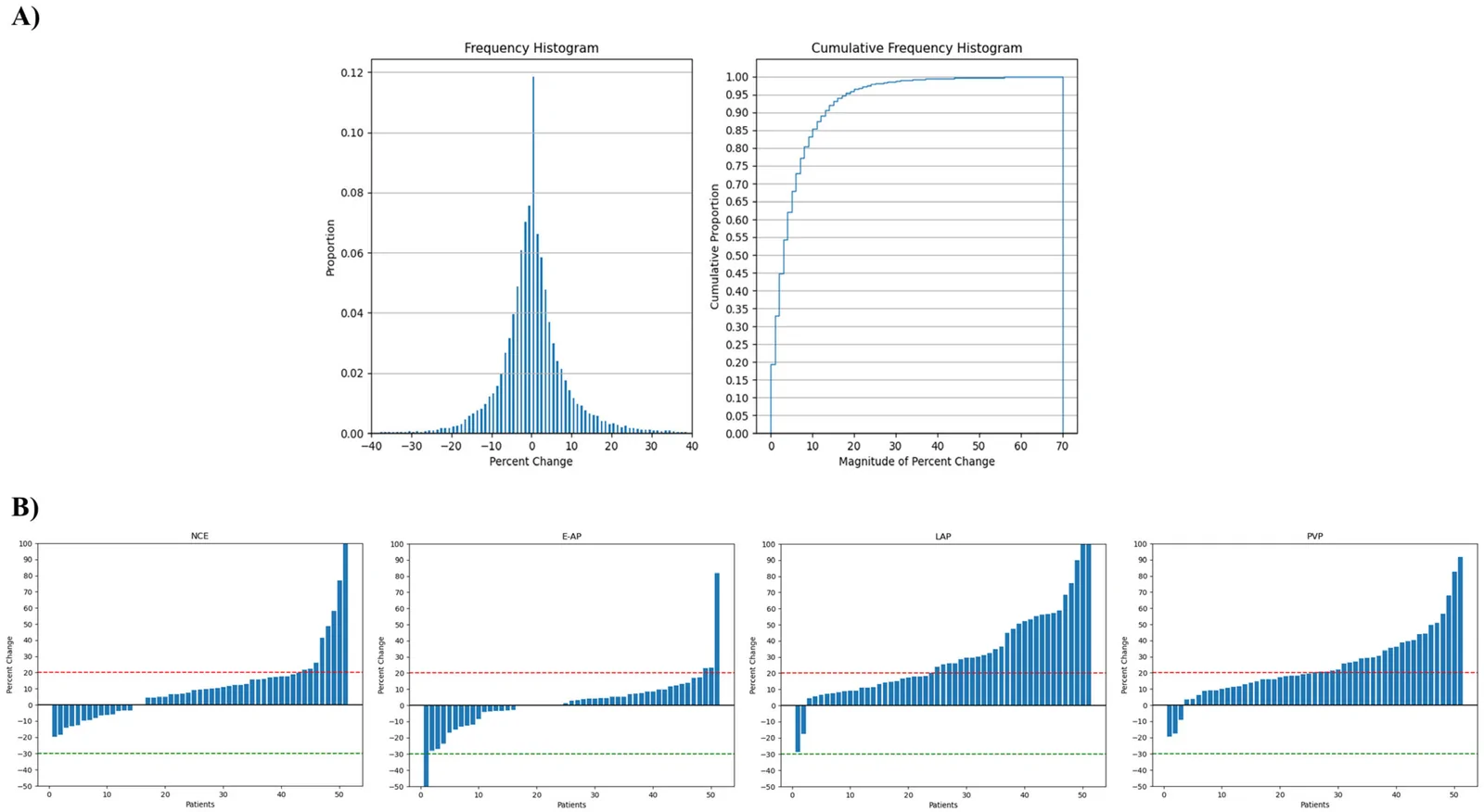
The percent differences between pairs of diameter measurements. (**A**) The histogram on the left displays the proportion of all measurement changes that fall within each one percent difference range. The largest proportion (about 12%) of percent changes in measurement were between 0 and 1 percent. The graph on the right displays the cumulative proportion of all measurement changes that fall within each magnitude of percent difference range. Approximately 95% of all percent changes in measurement fall within a magnitude of 20 percent. (**B**) These waterfall plots display the maximum value of percent change for each patient in different phases. In each phase, more of the maximum values are above the RECIST positive 20% threshold (red dashed line) for disease progression than the negative 30% threshold (green dashed line) for partial treatment response.

Overall, the mean lesion diameter was 32.9 ± 13.16 mm. The overall variability (as calculated per the CV) was found to be 5.11% (95% CI: 4.20–6.01%). The frequency distribution of the relative diameter changes between pairs of measurements is shown in Figure 3A. As shown in the figure, approximately 83% of these changes are within ±10%. About 3% of these changes meet the modified RECIST threshold for disease progression (at least 20% increase in diameter), whereas only 0.3% of these changes meet the modified RECIST threshold for treatment response (at least 30% decrease in diameter).

When analyzed according to phase, for the NCE phase, the mean lesion diameter was 32.85 ± 13.12 mm, with a variability of 4.69% (95% CI: 3.52–5.85%). For the E-AP, the mean lesion diameter was 32.54 ± 12.86 mm, and the CV was 4.55% (95% CI: 2.57–6.53%). For the L-AP, the mean lesion diameter was 33.05 ± 13.25 mm, with a variability of 5.82% (95% CI: 4.76–6.89%). Finally, for the PVP, the mean lesion diameter was 32.86 ± 13.28 mm, with a variability of 5.05% (95% CI: 4.21–5.90%).

**Figure 4:**
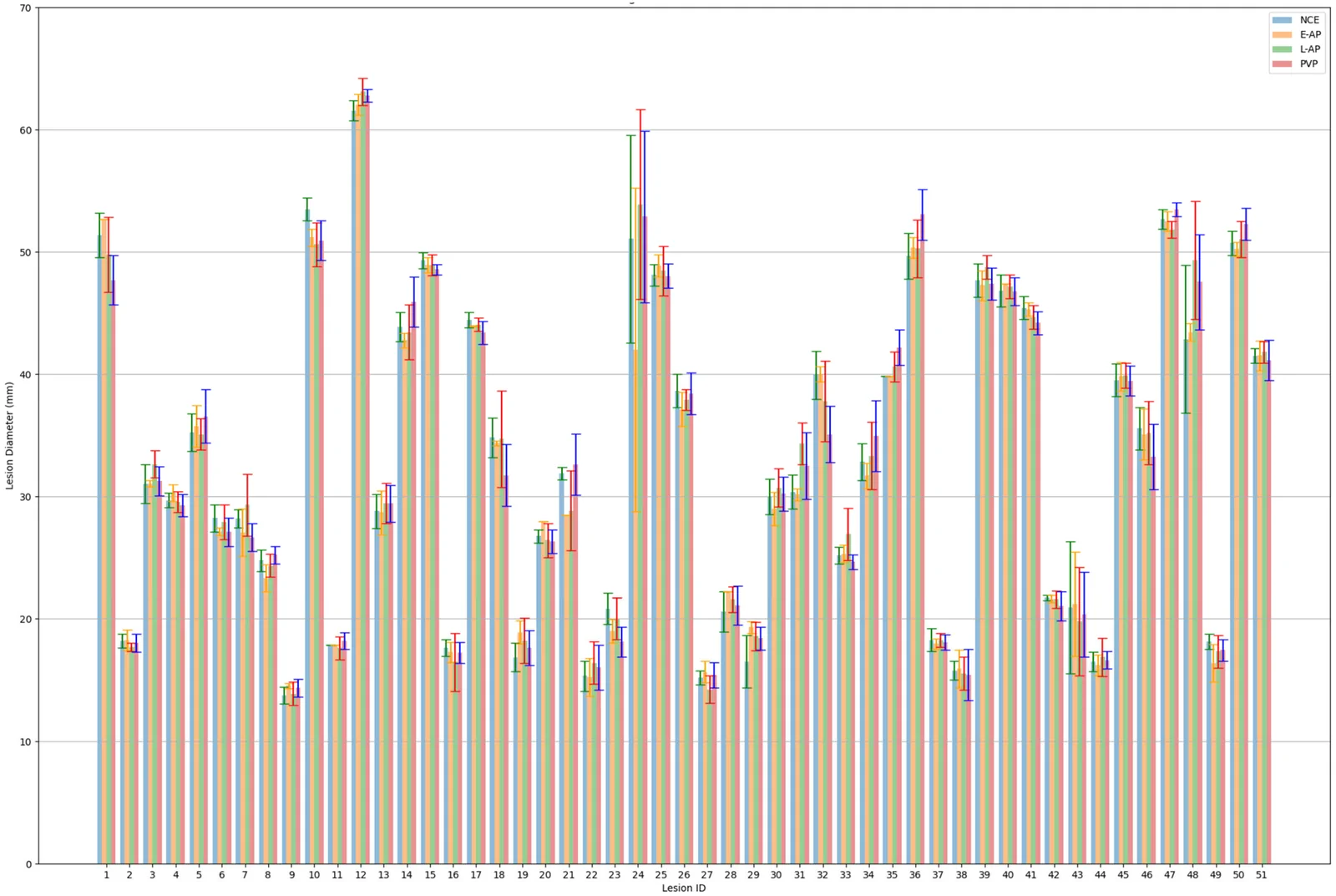
Average lesion diameter measurement by phase. The graphs above display the average lesion diameter measurement (mm) across all scans labeled a given phase for each patient. The error bars represent one standard error of uncertainty.

Figure 3B displays waterfall plots of the maximum relative change in diameter for each lesion across the four phases. Using the maximum percent changes for each patient in the NCE phase and E-AP, 15.7% (8/51) and 5.9% (3/51) of patients, respectively, would have met the modified RECIST threshold for disease progression. This proportion was much larger for the L-AP and PVP, where 52.9% (27/51) and 51.0% (26/51) of patients, respectively, would have met the same criteria. No patients met the modified RECIST threshold for treatment response in the NCE phase, L-AP, and PVP, and only one patient met the threshold in the E-AP. Figure 4 shows the average lesion sizes (mm) measured by phase for each patient. As shown in the graphs, there is no significant difference between lesion size measurements across the four phases given the overlap of the standard error bars for each lesion.

### 3.2. Variability of Diameter Measurement by Lesion Size

**Figure 5:**
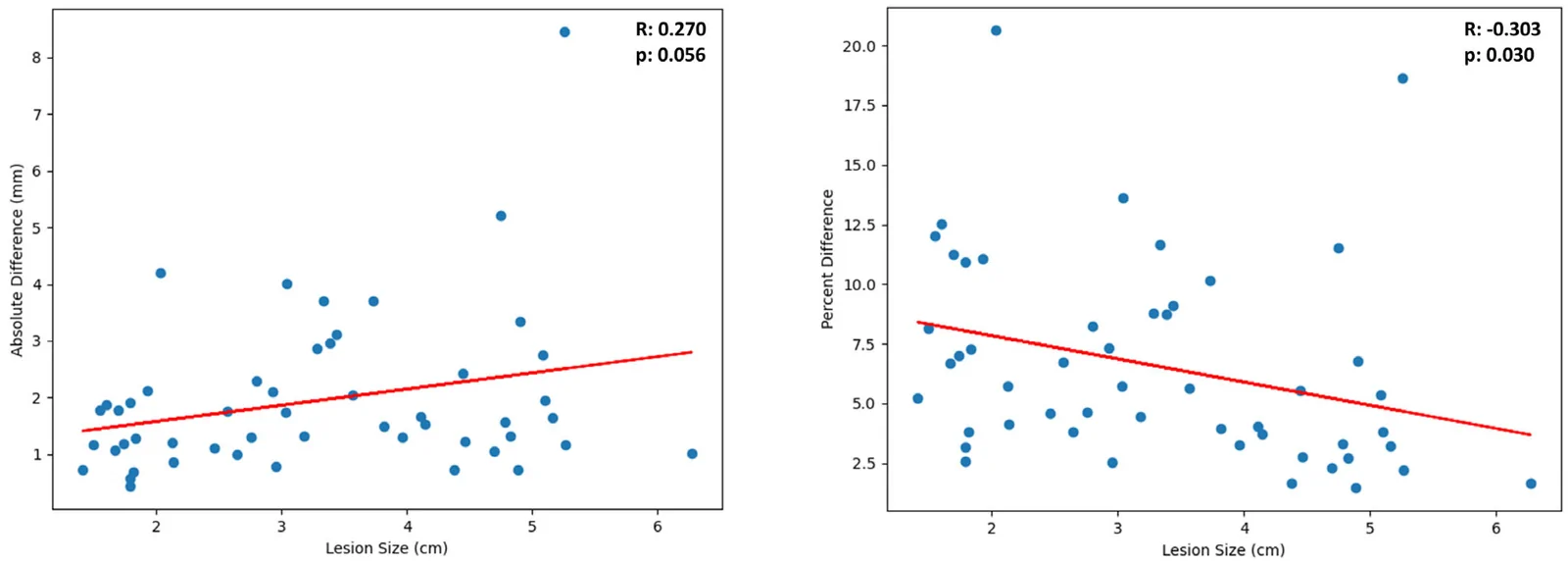
Correlation of percent difference with diameter measurements. The graph on the left plots the average magnitude of absolute difference between lesion measurements (mm) against average lesion diameter (cm) for each patient. Although the magnitude of the differences increases as lesion diameter increases, the correlation is borderline and not statistically significant. The graph on the right plots the magnitude of percent difference between lesion measurements against average lesion diameter (cm) for each patient. The decrease in percent difference as lesion diameter increases is statistically significant at α = 0.05.

The average lesion size for each patient across all phases was used to stratify all 51 lesions into four different size categories: 1–2 cm, 2–3 cm, 3–4 cm, and 5–7 cm. In each of these categories, there were 13, 10, 22, and 6 lesions, respectively. Table 3 displays the mean tumor size (cm), SD (cm), and the CV for each of the four size categories. An example tumor is shown for each category to demonstrate the range of potential measurement changes as calculated using the 95% limits of agreement with the standard deviation. Larger tumors tended to have larger magnitude measurement changes in centimeters (*p*-value = 0.056; Figure 5). In contrast, relative change was found to significantly decrease as tumor size increased (*p*-value = 0.030; Figure 5).

### 3.3. Variability of Density Measurement by Phase

**Figure 6:**
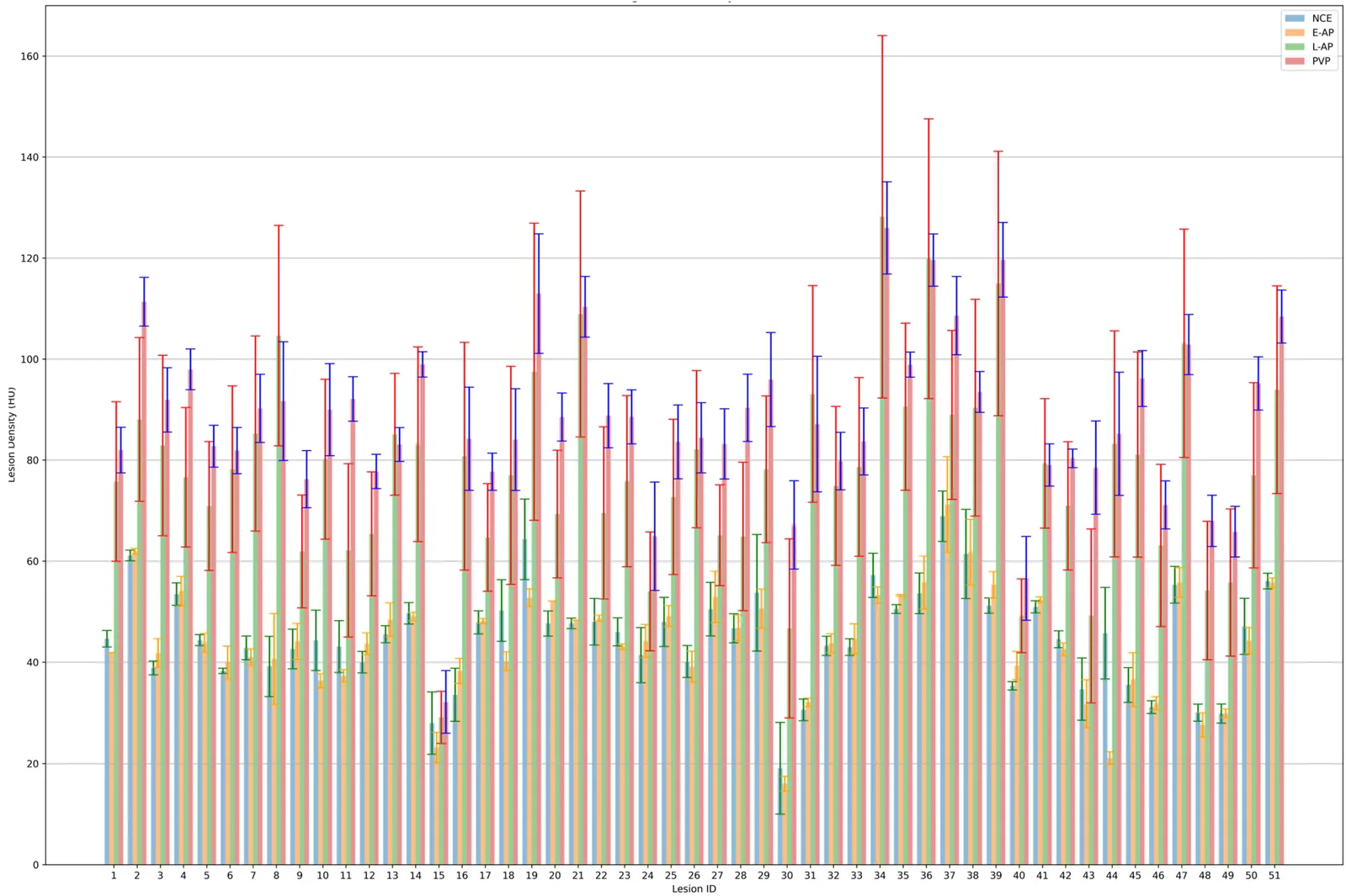
Lesion density across all phases. The graphs above display the average lesion density measurement (HU) across all scans labeled a given phase for each patient. The error bars represent one standard error of uncertainty.

The density of the lesions was found to have no significant change in the NCE phase and E-AP, followed by a significant increase in the L-AP and PVP, as demonstrated by the 95% confidence intervals for mean lesion density by phase: NCE (42.4–47.7 HU), E-AP (41.3–47.2 HU), L-AP (72.8–83.2 HU), and PVP (83.4–92.6 HU). This trend can also be observed in the lesion density graphs in Figure 6. The mean and standard deviation of lesion density by phase are reported in Table 4, along with the range of variability as calculated using the 95% limits of agreement with the standard deviation. The NCE phase, E-AP, and PVP exhibited comparable variability relative to the magnitude of lesion density, whereas the L-AP exhibited significantly greater absolute (±36.4 HU) and relative (±47%) variability. When observing across all phases, the lesion density was found to vary by as much as 54%.

## 4. Discussion

In this study, we assessed the impact of contrast enhancement on the variability of HCC lesion diameter and density measurements taken across CT scans of four different phases. The overall variability of lesion diameter measurement was about 5.11%. The vast majority of variations in tumor diameter (about 83%) fell within ±10%, and about 62% fell within ±5%. These proportions are similar to the findings of a previous study of lung tumor measurements taken on repeat CT scans [9]. However, this suggests that almost a fifth of all measurements would have a greater than 10% change due to variability from radiologist measurement across different phases alone. Thus, extra scrutiny should be paid to clinical trials that report changes within this range without consideration of other endpoints, particularly if attention was not paid to the phase of scans on which measurements were taken. This provides strong motivation to attribute any changes in tumor size that fall within 5% to variability alone in the absence of other clinical information, such as tumor necrosis, biomarker levels, or liver function tests.

Although the NCE phase and E-AP appeared to have smaller ranges of measurement with fewer patients meeting the modified RECIST for progressive disease (Figure 3B), nearly all major clinical practice guidelines endorse the use of CECT with the L-AP, the PVP, and a delayed phase (about 3–5 min after contrast injection) to diagnose and stage HCC [21,22,23,24,25]. Figure 3 shows that there was no statistically significant difference between average lesion measurement across the four different phases. When stratified by patient, large variability was typically consistent across the four phases, if present. Overall, our data suggest that the contrast enhancement phase does not significantly impact the variability of tumor diameter measurement in HCC.

One factor that does impact the variability in measurement is tumor size. There was a near-significant increase in absolute measurement difference as lesion size increased and a significant decrease in relative measurement difference as lesion size increased. In other words, the smaller the lesion, the more likely a relatively large difference in measurement due to variability alone. An example of this is displayed in Table 3, where smaller lesions of 1–3 cm in size could vary in measurement by over 20%. This reinforces the inclusion of absolute minimums in tumor growth for qualifying as “progressive disease”, such as the minimum 5 mm increase in the RECIST version 1.1 guidelines [2]. Possible modifications to consider in future response assessment criteria include increasing the percent thresholds for progressive disease for smaller tumors (<3 cm). The partial response threshold of a 30% decrease appears to maintain clinical significance at all size ranges, given that all size ranges of variability do not exceed it and that only 0.3% of all pairwise changes have met the threshold (10 times less frequently than for the progressive disease threshold).

As expected, lesion density was the image feature that varied the most across different phases. Overall, the CV for density measurement was 26.19% (24.66–27.72%). The 95% confidence intervals for mean lesion density by phase suggested that lesion density remained constant throughout the NCE phase and E-AP, with CVs of 9.62% and 7.58%, respectively. The density increased in the L-AP and PVP, with CVs of 22.84% and 7.83%, respectively, similar to the example shown in Figure 1. Most importantly, the lesion density was extremely variable in the L-AP compared to the other phases, with measurements ranging by about 50%. Physiologically, this can be explained by the flow of contrast from the aorta into the hepatic artery and eventually into the vasculature of the lesion itself. As a result, the specific timing of a lesion density measurement within the L-AP can significantly impact its reproducibility. For example, if a density measurement is taken at peak enhancement for a baseline scan and then towards the end of the L-AP on a follow-up scan, the decrease in tumor density could be falsely interpreted as a partial response to therapy when in fact it was due to intraphase variability from the physiologic distribution of contrast. Because the speed at which contrast distributes not only changes between patients but also within the same patient as liver physiology changes with disease progression or improvement, it can be difficult to select a narrow window within the AP for taking reproducible measurements. Thus, lesion density measurements should preferably be taken in the PVP over the L-AP to minimize variability and more accurately capture the real effect of treatment. It is important to note that phase selection should also be determined by optimal visualization of tumor characteristics, which in certain cases may require using the L-AP.

Moreover, our findings are consistent with prior studies that also highlight the importance of standardized scanning protocols in reducing tumor measurement variability for HCC and other hepatic malignancies. For instance, Martin et al. demonstrated that consistent arterial-phase timing significantly decreased inter-reader variability in assessing HCC lesions [26]. Similarly, Jones et al. reported that smaller lesions exhibit disproportionately higher relative variability, reinforcing the importance of considering lesion size when interpreting changes in tumor measurements [27]. These studies, alongside our results, underscore the need for uniform imaging guidelines in both clinical practice and research so that phase-dependent differences do not confound the accurate assessment of tumor progression or response.

The results are also in line with other quantitative imaging feature variability across different contrast enhancement phases. Previous studies have shown that differences in imaging phase significantly affect a proportion of features that are dependent on the density of segmented lesions [28]. The variability in imaging phase has also been reported to affect the performance of quantitative imaging features in predicting clinical outcomes [29,30].

Because only a single lesion was measured for each patient, this study is limited in its ability to analyze the variation in the measurement of multiple lesion diameters. As the variance should increase proportionally with the number of measured lesions, the standard deviation of such measurements should increase proportionally to the square root of that number. This suggests that for summed diameters (which increase proportionally by the number of lesions measured), the magnitude of standard deviation relative to the sum of diameter measurements should decrease. Simply put, there should be less relative variability when multiple lesions are measured, and the sum of their diameters is used as a criterion for response assessment. Another limitation of the study is that the lesion segmentations of a single patient were not entirely independent, as they were edited from copy–pasted contours from the initially segmented scan. However, the impact of this would be to decrease rather than increase measurement variability, keeping in line with the goal of providing the most conservative estimate.

## 5. Conclusions

The observed measurement variability across contrast phases reinforces the importance of following existing clinical guidelines, such as the recommendations for standardized multiphase CT protocols. By recognizing that measurements within a 10% range may be attributable to inherent variability, practitioners can avoid misclassifying borderline changes in tumor size as genuine progression or response. This can ultimately refine patient management decisions and improve the reliability of response assessment in clinical trials. Future iterations of response criteria might consider phase-specific thresholds or incorporate guidance on standardized scan timings to further mitigate these sources of variability, ensuring a more accurate reflection of true clinical changes.

## Figures and Tables

**Table 1:** Patient characteristics.

Number of patients	51
Age (years) ± standard deviation	54 ± 13
Male	45 (0.88)
Female	6 (0.12)
Cirrhosis cause Alcohol	9 (0.18)
Hepatitis B	45 (0.88)
Hepatitis C	1 (0.02)
HIV	0 (0.0)
Hemochromatosis	0 (0.0)
NASH	0 (0.0)
Unreported	1 (0.02)
Pathology **	
Well-differentiated HCC	3 (0.06)
Well-differentiated to moderately differentiated HCC	6 (0.12)
Moderately differentiated HCC	23 (0.45)
Moderately to poorly differentiated HCC	14 (0.27)
Poorly differentiated HCC	5 (0.10)

Note. ** data are expressed in percentages. Etiology of cirrhosis are non-mutually exclusive. NASH = nonalcoholic steatohepatitis, HIV = human immunodeficiency virus.

**Table 2:** Acquisition and reconstruction parameters of imaging data.

Vendors	Model	X-Ray Tube Current (mA)	Exposure Time (ms)	kVP	CTDVol (mGy)	Reconstruction Kernel	Slice Thickness (mm)	Pixel Spacing (mm^2^)
Toshiba	Aquillon	50–250	500–4000	120	9.6–14.4	FC02	2, 5, 8	0.56 × 0.56–1.0 × 1.0
						FC04		
						FL03		

**Table 3:** Variability of lesion diameter measurements by average lesion size.

Size of Tumor (cm)	Coefficient of Variance (CV)	Standard Deviation (cm)	Example Tumor		
			Size (cm) *	Range as a Result of Variability (cm) **	% Change as a Result of Variability
**1–2**	5.80% (4.50–7.10%)	0.15	1.7	1.4–2.0	±18
**2–3**	5.60% (2.66–8.54%)	0.34	2.5	1.9–3.1	±23
**3–5**	4.65% (3.39–5.90%)	0.65	4.0	3.5–4.5	±12
**5–7**	4.45% (−0.87–9.77%)	0.46	5.4	4.6–6.2	±14

* mean tumor size (cm) for size category; ** 95% confidence interval based on standard deviation provided for size category.

**Table 4:** Variability of lesion density measurements by contrast enhancement phase.

Phase	Coefficient of Variance (CV)	Standard Deviation (HU)	Example Tumor		
			Mean Density (HU)	Range as a Result of Variability (HU)	% Change as a Result of Variability
**All**	26.19% (24.66–27.72%)	15.57	76.41	34.8–118.0	±54
**NCE**	9.62% (7.15–12.09%)	9.73	45.03	36.4–53.6	±19
**E-AP**	7.58% (6.08–9.09%)	10.59	44.25	37.1–51.5	±16
**L-AP**	22.84% (21.48–24.20%)	18.93	77.96	41.6–114.4	±47
**PVP**	7.83% (6.76–8.89%)	16.68	88.01	73.6–102.4	±16

## Data Availability

The data presented in this study are openly available in FigShare at 10.6084/m9.figshare.28266551.
